# Effect of a digital dietary intervention on diet quality in young adults in Norway – The PREPARED randomised controlled trial

**DOI:** 10.1186/s40795-026-01278-4

**Published:** 2026-02-21

**Authors:** Erlend Nuland Valen, Andrew Keith Wills, Anine Christine Medin, Frøydis Nordgård Vik, Dagrun Engeset, Lorentz Salvesen, Elisabet Rudjord Hillesund, Nina Cecilie Øverby

**Affiliations:** Department of Nutrition and Public Health, Centre of Lifecourse Nutrition, University of Agder, Postbox 422, Kristiansand, 4604 Norway

**Keywords:** Preconception, Diet quality, Micronutrient adequacy, Randomised controlled trial, Norway

## Abstract

**Background:**

Nutritional status and lifestyle during the preconception phase, the months and years before conception, may have substantial impact on the health of the next generation. Diet quality during these years has been a neglected in previous dietary assessments.

**Methods:**

The PREPARED study is a digital intervention with a randomised controlled trial design with the aim to assess its effectiveness on diet quality and micronutrient adequacy. Individuals with a Norwegian identification number without biological children aged 20–35 were recruited using social media platforms. Data was collected digitally using a dietary screener which was recoded into a diet quality score (DQS) and a 24-hour recall recoded into binary variables to compare adherence to the average requirement defined by the Nordic Nutrition Recommendations 2023. The intervention group received access to a digital resource where all components were created using the Determinants of Nutrition and Eating framework (DONE), with new weekly content across 6 months.

**Results:**

From the total sample of 1374, the follow-up sample consisted of 186 participants (13.5%) (Control: 99, Intervention: 87) on DQS, and 150 (10.9%) on micronutrient adequacy. The mean difference between intervention and control in total diet quality score was + 1.0 (IQR − 3.0, 4.9) (adjusted *p* = 0.63). The results showed no improvement in diet quality or change in proportion of participants reaching average requirements between the intervention and control group.

**Conclusions:**

We found no evidence towards having a healthier diet in any of the groups at the end of the trial, and there were no differences in proportions reaching average requirement for selected micronutrients. The substantial loss to follow-up might have had a significant impact on the results. Improving or developing strategies for recruitment and keeping retention rates is an important area for future intervention studies.

**Trial registration:**

ISRCTN44294662 (04.02.2021).

**Supplementary Information:**

The online version contains supplementary material available at 10.1186/s40795-026-01278-4.

## Introduction

Research suggests that the nutritional status and lifestyle of both men and women before conception, the preconception phase, has a substantial impact on birth outcomes in their children [1–3] . In 2023, a total of 134 million babies were born world-wide, highlighting the great potential improved preconception diet might have on the next generation [4]. The Lancet series from 2018 on preconception health identified potential ways towards improving preconception diet in the population [5–7]. From a public health perspective, the preconception period can relate to the entire fertile age range. Some cohort studies suggest that dietary patterns from up to 3 years before pregnancy may have an impact on several health outcomes [6]. An optimal preconception diet is considered following the national dietary guidelines, with a specific additional recommendation for women to supplement folic acid at least three months before conception [8, 9]. In Norway, there is also additional recommendations of supplementing vitamin D, vitamin B12, Iodine and Omega-3 fatty acids during this period [9].

In a review by Raghavan et al. [10] Mediterranean preconception dietary patterns, often characterised with high intakes of fruit, vegetables, whole-grain, fish, vegetable oil, nuts and legumes, were associated with lower risk of hypertensive disorders of pregnancy and preeclampsia. Li et al. [11] found similar associations with higher adherence using various scoring methods to assess diet quality.

Recent research from Wills et al., and Van Lippevelde et al. has even identified associations between diet in adolescence and later adverse maternal and child outcomes [1, 12]. Salvesen et al. showed that better knowledge about the concept of preconception health was associated with higher diet quality scores [13]. However, research suggest that knowledge is lacking about preconception health [14–16]. Having higher knowledge about nutrition was associated with having higher diet quality, implying that increasing the knowledge about nutrition in the general population might increase diet quality [16].

Studies assessing diet in young adults results imply that there are several aspects in their diet that needs improving. In Europe, only 12.5% of those aged over 18 consume five fruit and vegetables a day [17], and men are more likely to report no daily consumption of fruit or vegetables [18]. Similarly, research on Norwegian students suggest that especially their fruit and vegetable intake is far lower than the recommendations, and results from the recently published Norkost 4 study from 2022 show similar results [19]. Adherence to Norwegian guidelines has been assessed based on the national Norkost 4 study, showing that 14% of the total sample aged 18–80 years adhered to guidelines on fruits, berries and vegetables, and an average adherence of 21% across all dietary guidelines [19], showing great potential for improvement. The highest adherence was identified as reducing intake of red meat with 40% meeting the guidelines of eating less than 350 g/week [19].

The PREPARED study is a randomised controlled trial (RCT) evaluating the effect of a digital dietary intervention promoting healthy dietary behaviour on knowledge and skills regarding diet, and improving health-related quality of life and preconception diet [20]. The aim of this article was to estimate the effect on the primary outcome of the PREPARED study – the effect of the intervention on preconception diet, including intake of micronutrients.

## Methods

### Study design and population

The PREPARED project is a nationwide randomised controlled trial of a digital dietary intervention with long-term follow-up of up to 20 years [20]. The study adheres to CONSORT guidelines. The intervention period lasted for 6 months and aimed to increase knowledge and skills regarding diet and food preparation, leading to a healthier preconception diet and improved quality of life [20]. Recruitment took place from August 2021 to December 2022 through adverts on popular Norwegian social media platforms such as YouTube, Instagram, Snapchat and Facebook after recommendations from the university’s communication department. Biweekly meetings were completed to assess which platform was most effective to redistribute funds for advertisements. Eligibility criteria were native speaking men and women aged 20 to 35 years with a Norwegian identification number [21], without biological children, internet access and an email address.

Potential participants were directed to a website administrated by the University of Agder with information about the project, and with a link from where they could register and start the baseline questionnaire. We used the self-service online form “Nettskjema”, developed at the University of Oslo [22], to digitalize and host the questionnaire. The questionnaire included background questions like age, gender, education, questions about physical activity and sedentary behaviour, tobacco usage, quality of life, knowledge about Developmental origins of health and disease, and a food screener (see Diet quality score).

Following the completion of the questionnaire, an invitation was sent to complete a digital self-administered 24-hour recall (24 h) called myfood24 [23]. A second 24 h invitation was sent two weeks later at an unannounced time, although participants had been informed that a follow-up would occur. Participants were randomised either after completion of the second 24 h, or 10 days after it was sent out if incomplete. Participants who completed only the questionnaire and not the 24 h were excluded from randomisation and the intervention. We used automatic block randomisation with block sizes between 2 and 8 constructed by a person external to the project to ensure correct randomisation regardless of sample size. The intervention group got access to a digital resource for 6 months. Upon randomisation, the control group received a reminder of the importance of continued participation in the study but were not provided with any additional information. After the intervention period was finished the same questionnaire was sent except for background questions, and thereafter two 24-hour recalls with two weeks in between.

### Diet quality score

Diet quality was assessed according to degree of adherence to Norwegian nutrition and food-based guidelines using a questionnaire with a diet quality score (DQS) developed at the University of Agder called MyFoodMonth 1.1 [24]. The food screener contained 33 items where intake was assessed using a scale with ten categories ranging from never to six or more times a day. These 33 items were then aggregated into ten categories as described by Salvesen et al. [24].

The ten score components were chosen to reflect all aspects of the dietary guidelines in Norway in 2023 [25] and closely resembles the WELL diet score [24, 26, 27]. Each component is scored from 0 to 10 points based on degree of adherence to the specific recommendation [26]. The score components comprise vegetables, fruit, whole grain, fish, legumes, unsalted nuts and seeds, red and processed meats, salty snacks, sugar-sweetened beverages (SSB) and sugary foods. The healthy components vegetables through unsalted nuts and seeds gave high scores on high intake, while the unhealthy components red and processed meats through sugary foods gave high scores with low consumption. For instance, an intake of 2–3 fruit per day will qualify for a score of 10 points as that is defined as meeting the guidelines, but you would not get a higher score if you consumed 4–5 or ≥ 6 per day. For a detailed description of the diet quality scoring and distribution of participants at baseline, see supplementary table S1.

A compiled score based on the ten components was used to create a total diet quality score (DQS) which ranged from 0 to 100 points.

### Micronutrient adequacy

Micronutrient intake was assessed using a validated digital 24HR [28–30]. The intake was compared to age- and gender specific Nordic Nutrition Recommendations from 2023 (NNR). Myfood24 is a digital dietary assessment tool which can be used as a food diary, as well as a 24-hour recall [23]. The Norwegian version developed at UiA was used in this project [31].

Participants registered all foods and beverages consumed by selecting items from the database, and were encouraged to use the comment section to document any use of dietary supplements or food items they could not find. All comments were reviewed manually by the researchers after the intervention was complete. The correct food items were either identified and added to each respondent’s registration, or when necessary, substituting with appropriate alternatives. All supplement use was registered manually by the researcher.

We created a set of binary variables (See supplementary table S2) derived from micronutrient intake indicating whether a person was meeting Nordic Nutrition Recommendations, or not. When assessing adherence to guidelines, it is recommended to use the average requirement as described in the NNR [32]. The micronutrients assessed for adherence was vitamin D, folate, iron, calcium, iodine, vitamin B12 and vitamin C as these are highly relevant for preconception health [6, 33–35].

### The PREPARED intervention

The intervention component was a web-based digital resource designed using WordPress [36]. Given that 99% of Norwegian adults under 44 use the internet daily [37], a digital intervention is accessible to almost the entire population. The intervention included 15 articles, 26 e-mails with links to short messages, and 36 recipes.

The articles contained information about basic food preparation skills, meal habits, different core dietary components and nutrients, as well as an introduction to what preconception means and what it entails for their own health and future children. All participants had access to all the articles from the beginning of the intervention.

The e-mails with links to short messages were based on an individual path of emails with the intention to direct the participants back to the website and give gentle nudges to promote healthy dietary behaviour. In total, 26 messages were sent one week apart, each with a unique subject which they could read more about on the website. The participants did not have access to these articles from the beginning but were gradually given access to the complete content. Within each email, a link to a relevant recipe was also provided.

In total, 36 recipes were created that emphasised the use of healthy ingredients prepared from scratch, while still requiring limited skill and time. Although they were not linked in the weekly messages, seven ‘healthier’ dessert alternatives among the recipes were provided as well as one healthier snack alternative in the form of toasted chickpeas.

All components within the website were decided upon using the Determinants of Nutrition and Eating framework (DONE), including food habits and beliefs, food and nutrition knowledge, skills and abilities, health cognition, eating regulation and self-regulation [38]. All content was in line with Norwegian dietary recommendations and relevant information, including the triple dividend of eating healthy, recipes with instructions, meal planning and cooking healthy meals [20].

### Analysis

The analysis was conducted on complete cases using an intention-to-treat approach. Two samples were used to analyse the DQS and micronutrients. To explore differences in diet quality scores, data from all participants who completed the dietary screener were included in the analysis. For the analysis of micronutrient intake, we restricted the dataset to those who had completed the first 24 h to maximise sample size and statistical power.

The sample was described with respect to demographic and other related characteristics at baseline using appropriate summary statistics. This was first done according to randomisation group in all participants randomised and in the analysis sample. Then, in the entire eligible sample (those that completed the baseline questionnaire) and those lost to follow-up to check for selection bias and internal validity of the between-group comparison.

The primary dietary outcomes were the total diet quality score (0 to 100 points), and energy adjusted (per 10 MJ) micronutrient adequacy from diet and supplements (calcium, folate, iodine, iron, vitamin B12, C and D) recoded to a binary variable to indicate if the participant met the average requirement (AR) from the NNR or not. Regression models were used to estimate mean difference in total DQS between groups, and the odds ratio of reaching the AR comparing the intervention versus the control group.

Two sets of models were fitted to estimate the intervention effects, first an unadjusted and second, a model adjusting for education which was the only variable with significant difference between groups. The adjusted models were estimated as a check of the internal validity of the study, acknowledging the fact that there was a high loss to follow up which may have compromised the randomisation. For the total diet score outcome, regression models were used to estimate differences in diet score between groups.

The secondary outcomes were the ordinal diet quality score components (vegetables, fruit, grains, SSB, legumes, nuts, red meat, fish and salty snacks) and continuous measures of micronutrient intake (energy adjusted and including supplement use).

Effects on the ordinal diet quality score were estimated using ordinal logistic regression, the purpose was to assess whether the intervention had an effect across the distribution of dietary food components. See supplementary table S1 for a description of how these variables were derived.

Effects on the continuous micronutrient intakes were estimated via regression on the log scale. The purpose of this analysis was twofold. First to test whether the intervention shifted the distribution of any of the micronutrient intakes. Second, to provide an insight into the effect of measurement error on our findings regarding micronutrients – these analyses give unbiased estimates in the presence of non-differential measurement error which is a less strict assumption than the analyses of the primary meeting micronutrient AR outcomes [39].

To further understand measurement error, we also estimated usual intake at baseline and follow up using information from the sample that completed two 24HR [40] (more details and results are in the supplementary material).

SPSS v29.0.0.0 and Stata SE v18.5 was used for all analysis.

## Results

### Sample description

**Fig. 1:**
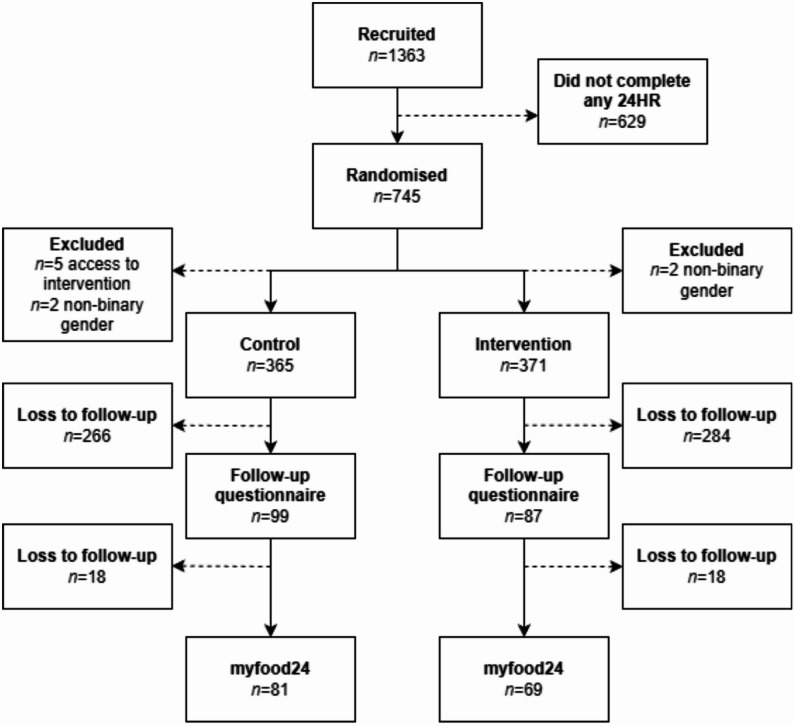
Flow-chart of recruitment, exclusion and loss of participants

A flow-chart of participants is presented in Fig. 1. From 1363 eligible participants that completed the baseline questionnaire, a total of 745 (54.7%) participants completed at least one 24-hour recall and were randomised to either a control group or an intervention group. After the intervention period (6 months) was completed for all participants, 5 participants were removed from the control group because they stated that they had access to the website, along with 4 who identified as a non-binary gender. Data from Google Analytics showed that the intervention group spent an average of two minutes on the web site each time they used it. One-hundred and eighty-six (13.5%) completed the follow-up questionnaire, and 157 (11.5%) completed at least one 24-hour recall, while 117 completed both 24-hour recalls (8.6%).

Table 1 describes the sample at baseline, in the total group, the group at the point of randomisation, the group lost to follow-up, and the remaining participants at 6 months follow-up. Age was similar in all four samples with an approximate age of 27 years. In total, 88% were women, and the proportion of men were lower among those that remained in the study, decreasing from 10% to 5% in the final sample. The level of education was quite high in all samples with 77% having higher education, which also increased in the sample at six months follow-up to 83% and 87% in control and intervention group respectively. From the same sample of six months follow-up, the control group had 49% with more than 4 years of higher education, while the intervention group had 33%. Baseline total diet quality scores (DQS) were approximately 60 points across all samples. Apart from education, the control and intervention group appeared comparable in terms of baseline characteristics, and as such, only education was adjusted for in the second set of models as a check of internal validity.

**Table 1:** Characteristics of the sample by group at baseline, statistics are presented for the entire sample, those randomised, those lost to follow-up, and those analysed

**Baseline variables**	**Total** ***n*=1363**^**(a)**^	**Randomised participants (*n*=736)**		**Lost to follow-up (*n*=627)**^**(b)**^	**Analysis sample (*n*=186)**^**(c)**^	
		**Control** **(*n*=365)**	**Intervention ** **(*n*=371)**		**Control** **(*n*=99)**	**Intervention ** **(*n*=87)**
Mean age (SD)	27.1 (3.9)	27.0 (4.0)	27.3 (3.8)	26.9 (3.9)	27.3 (3.8)	27.5 (3.7)
Female N (%)	1207 (88.6)	330 (90.4)	334 (90.0)	543 (86.6)	94 (94.9)	82 (94.3)
Height, m (SD)	1.70 (0.08)	1.69 (0.07)	1.70 (0.08)	1.70 (0.08)	1.68 (0.07)	1.70 (0.08)
BMI (SD)	24.7 (5.0)	24.4 (4.7)	24.7 (4.6)	24.9 (5.4)	23.9 (4.4)	24.3 (4.2)
Education, n (%)^(d)^						
No higher education	320 (23.5)	79 (21.6)	86 (23.2)	155 (24.7)	17 (17.2)	14 (16.1)
Higher education (≤4 years)	526 (38.6)	129 (35.3)	147 (39.6)	250 (39.9)	35 (34.3)	44 (50.6)
Higher education (>4 years)	517 (37.9)	157 (43.0)	138 (37.2)	222 (35.4)	48 (48.5)	29 (33.3)
Diet quality score (IQR)						
Total diet score (SD)	59.1 (13.9)	60.2 (14.0)	59.3 (13.3)	58.4 (14.2)	60.4 (13.6)	61.9 (13.9)
Vegetables	8 (6, 9)	8 (6, 9)	8 (6, 9)	8 (6, 9)	8 (8, 9)	8 (6, 9)
Fruit	6 (4, 10)	6 (4, 10)	6 (2, 8)	6 (4, 10)	6 (2, 10)	6 (4, 10)
Whole grain	8 (6, 10)	8 (8, 10)	8 (6, 10)	8 (6, 10)	8 (8, 10)	8 (8, 10)
SSB	9 (6, 10)	9 (6, 10)	9 (6, 10)	8 (4, 10)	8 (4, 10)	9 (6, 10)
Sugary foods	4 (1, 6)	4 (1, 6)	4 (1, 6)	4 (1, 6)	4 (4, 6)	4 (1, 6)
Legumes	4 (2, 6)	4 (2, 6)	4 (2, 6)	4 (2, 6)	4 (2, 6)	6 (2, 6)
Unsalted nuts and seeds	4 (2, 6)	4 (2, 6)	4 (2, 6)	4 (2, 6)	4 (2, 6)	6 (2, 6)
Red and processed meats	4 (2, 8)	4 (2, 8)	4 (2, 8)	4 (2, 8)	6 (4, 10)	6 (4, 8)
Fish, fatty, lean and spread	9 (7, 10)	9 (7, 10)	9 (7, 10)	9 (7, 10)	10 (7, 10)	9 (7, 10)
Salty snacks	6 (4, 8)	6 (4, 8)	6 (4, 8)	6 (4, 8)	6 (4, 8)	6 (4, 8)
Micronutrient adherence, %	*n*=733	*n*=363	*n*=370	**-**	*n*=80	*n*=69
Calcium	53.13	54.3	57.9	**-**	53.8	55.1
Folate	54.0	55.0	53.0	**-**	55.0	62.3
Iodine	43.0	43.7	42.4	**-**	40.0	52.2
Iron	60.3	61.5	59.1	**-**	65.0	69.6
Vitamin B12	70.2	69.3	71.1	**-**	68.8	78.3
Vitamin C	59.3	58.7	59.8	**-**	62.5	68.1
Vitamin D	33.4	30.3	36.5	**-**	31.3	36.2

^(a)^Includes all those recruited and eligible

^(b)^Did not complete any 24-hour recalls

^(c)^For the analysis of dietary outcomes, for micronutrient outcomes *n* = 150

^(d)^Education of up to four years equals a bachelor’s degree, while over four years equals a master’s degree or higher

### Diet score

There was little evidence to suggest that the PREPARED intervention had any effect on total diet quality score or meeting any of the dietary recommendations for each diet quality component. The mean total diet score in the control and intervention group was 60.8 and 61.1 points respectively (mean difference between groups: +0.4; 95% CI -3.6, 4.3; *p*-value: 0.86). Adjusting for educational level did not alter this finding (Supplementary table S3).

**Fig. 2:**
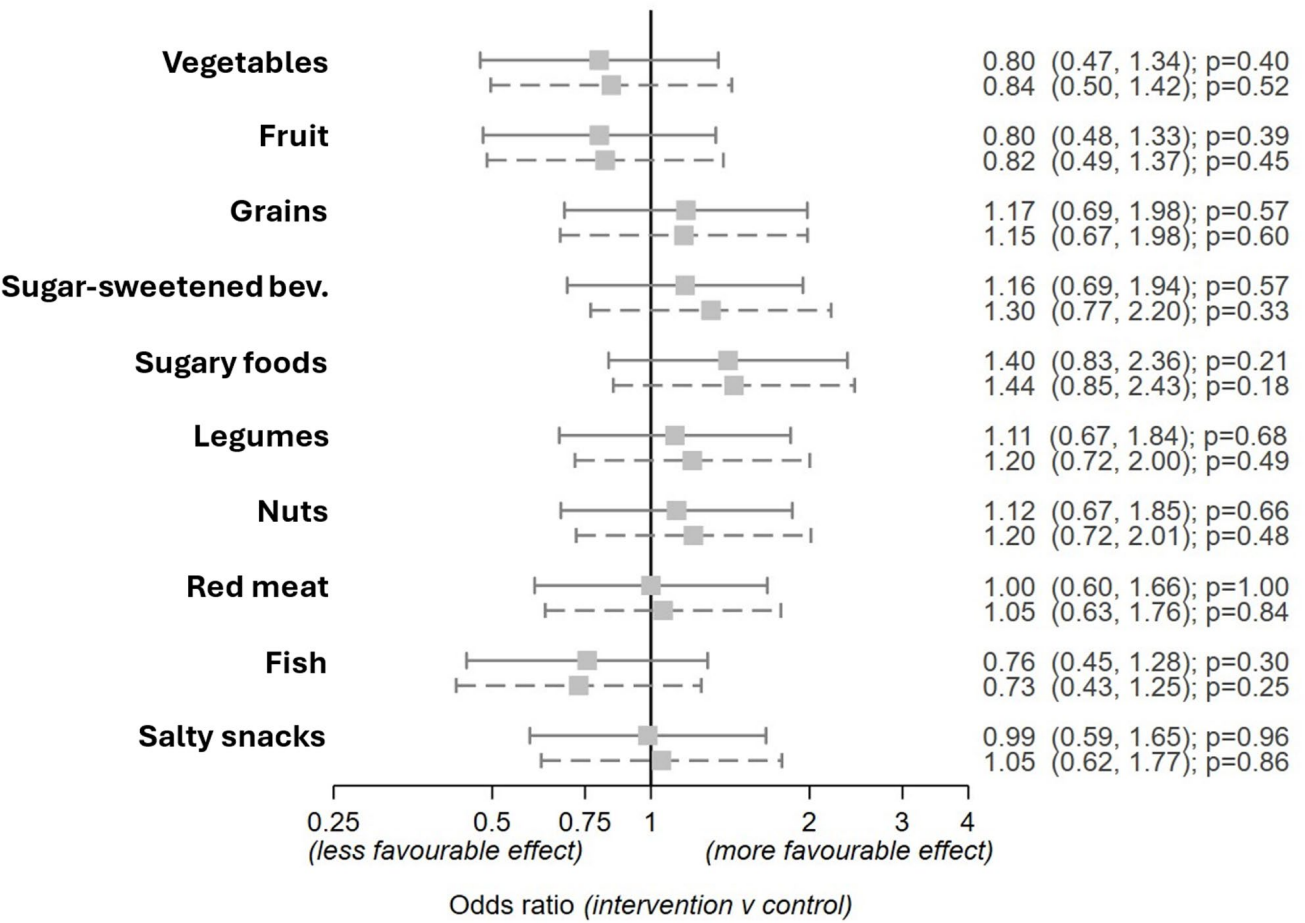
Estimated effects of intervention on odds of more favourable intake* of diet components comparing intervention versus control group. The solid lines are the unadjusted effects and the dashed adjusted for education. The odds ratios and 95% CI are listed on the right-hand side of the plot. *Higher diet quality score (where SSB,* sugary foods*,* red meat and salty snacks give high scores on low intake)*

Figure 2 plots the estimates for the intervention effect on each component of diet. There was little suggestion of a difference between the intervention and control groups at 6 months for any individual components of diet.

### Micronutrients

**Fig. 3:**
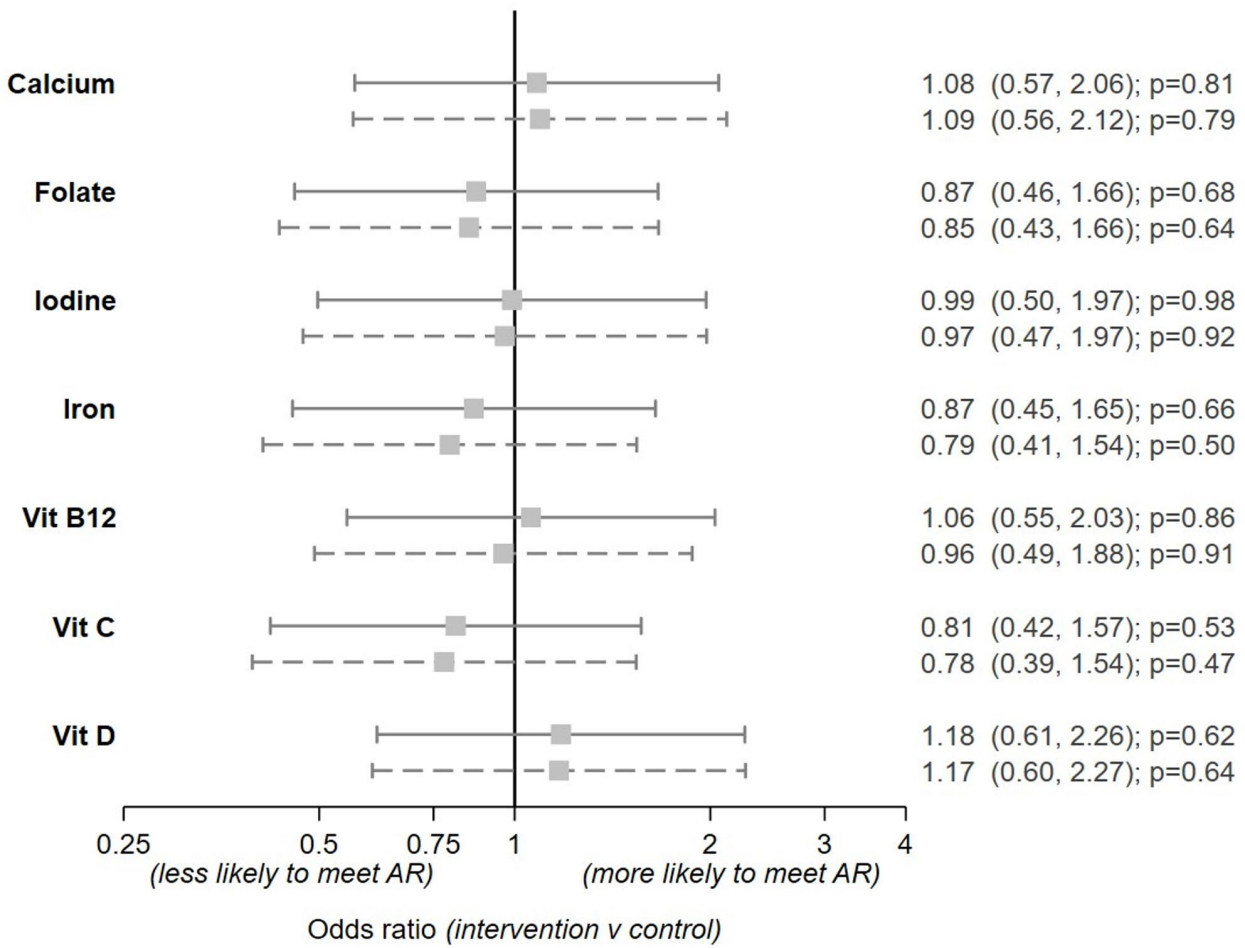
Estimated effects of intervention on odds of likelihood of reaching average requirement* comparing intervention versus control group. The solid lines are the unadjusted effects and the dashed adjusted for education. The odds ratios and 95% CI are listed on the right-hand side of the plot. *As described in the Nordic Nutrition Recommendations 2023 (29)

Figure 3 plots the estimates for intervention effect on micronutrient adequacy based on the first 24 h (*n* = 158). There was little evidence for effect of the intervention on meeting average requirements for any of the micronutrients compared. Adjusting for education also made no difference to the findings.

Supplementary figure S2 shows the results from the secondary analysis of continuous micronutrient intake. These analyses did not produce any evidence to suggest that the intervention had any effect on proportional shifts across the entire intake distribution.

Estimates of usual micronutrient intakes and proportions meeting Norwegian AR based on regression calibration using the sub-sample with two repeated 24 h are shown in the supplementary material. Within-person day-day variability was larger than between-person variability, usual intake distributions were hence shrunk substantially compared to the distributions based on a single 24 h. However, this had little effect on the general findings (more details available in the supplementary material).

## Discussion

To the best of our knowledge, PREPARED is the first study to trial a digital intervention to promote a healthy preconception diet [41]. This paper reports on the effectiveness of the PREPARED study on diet quality and micronutrient adequacy. In our population of fairly high educated young adults, we could find no evidence that the 6-month intervention had any effect on overall diet quality, components of diet or proportion reaching average requirements of micronutrient intake immediate post-intervention.

The lack of effect on overall diet quality might be a consequence of the small sample size post-intervention. The relatively small sample size makes it more difficult to detect small differences [42]. For dietary data based on single recall where the target of inference is usual intake this problem is also amplified by the large day-day variation and/or measurement error as we demonstrated in our data [40, 43, 44]. A digital intervention can potentially reach a wide audience, but the size of effect might be limited [45] as behaviour change is dependent on the participants’ own willingness to use the website. Other interventions have used active procedures to create behaviour change, such as online personal coaching [46] and conversational agents [47]. Others primarily focuses on women that are actively trying to conceive, or are already pregnant, which is considered a highly motivated group with very high retention rates [48, 49].

Regarding use of the website, we do not have similar data from other studies to compare with, making it difficult to evaluate level of engagement. It seems, however, that two minutes was not enough time spent to encourage behaviour change that was detectable in our analyses. In addition, we do not know if everyone used the website, which could also influence our results. The lack of ability to track participant engagement with the digital resource has limited the opportunities to conduct analyses on different groups with different levels of engagement. Due to the small sample size, post-intervention between-group differences would need to be relatively large to prove effect of the intervention.

A report from 2021 showed that 46% of the Norwegian population uses dietary supplements daily [50]. Therefore, we collected information on supplement use as it was assessed as a central part of the Norwegian diet. The decision to assess micronutrient adequacy as binary variables was made for its public health and nutritional relevance but also to mitigate the wide range of micronutrient intakes across our sample. Many of the supplements that were consumed contained much higher amounts than the recommended dosage and would skew the sample. By using a binary variable, the outliers would be given the same values regardless of how extreme the intakes were. On the other hand, a potential improved intake might be difficult to detect among the high proportion of participants meeting AR to begin with.

This intervention was directed towards adults aged between 20 and 35 years regardless of whether they have an intention to have children or not, reaching two of the four preconception groups defined by Barker et al. [7]. Including this age range allowed us to capture individuals across different stages of life. Although the preconception group is defined as adult being above 16–18 years, most in this age group still attend public schools and/or live at home with their parents, which might impact diet quality and lifestyle choices and their possibility to change them [20]. Individuals over the age of 35 were excluded due to ethical considerations, aiming to reduce the risk of any emotional burden of involuntary childlessness due to age-related factors. The average age of first time parents in Norway in 2023 was 30.3 years for women, and 32.3 years for men [51], and the goal was to reach a younger group than this to increase the chance of pregnancy in the future.

The high proportion of well-educated participants may have impacted the results as a higher education level is strongly associated with better physical and mental health, as well as life expectancy [52], and makes the sample less representative. As a well-educated group the diet quality scores are quite high at baseline, creating less opportunity to improve diet. In a population with a lower educational level, and possibly lower diet scores we might have seen different results. Broader reach, including groups with lower socioeconomic status (SES) might also give different results as lower SES is associated with lower diet quality [52] and a greater potential for benefit of the intervention.

In the Lancet series on preconception, Barker et al. stated that there is a need for robust and context-relevant trials of preconception nutrition and health behaviour interventions on a large scale [7]. In a systematic review from 2023, O’Connor et al. found ten preconception interventions with different delivery methods [41]. The most commonly used method was face-to-face or telephone counselling [41]. Only one intervention used a website as the delivery method, showing that there is unexplored potential in this area [53]. This novel approach might need some adjustments to reach the people who really need it, to motivate the group towards using the website, and trigger a positive behaviour change.

The main strength of this study is the randomised controlled trial design which gives the opportunity to draw causal connections [54]. We assess the randomisation to be of high quality as researchers were blinded to the randomisation and assessment of outcomes [20, 55]. However, with the final sample being quite small, there is a chance for random imbalances between groups although adjusted models produced similar finding. The generalisability of the findings might be limited to women with relatively high education and must be interpreted with caution.

Despite efforts towards keeping retention rates, and the choice of randomising after one 24-hour recall, we had much higher drop-out rates than anticipated. Loss to follow-up bias may explain some of our results, although the drop-out did not appear to be selected by certain characteristics differentially between groups.

By using both 24 h and dietary screener to assess diet, we hoped to gain a better understanding of different aspects of young adults’ diet. Using a 24-hour recall is one of the preferred ways of examining intervention effects [56], and one of the major upsides of using a digital 24-hour recall should be that it reduces the burden for both participant and researcher [57]. However, in our experience, it was a major burden for the participants, because the main drop-out sources came from the steps containing 24 h. The 24 h is also limited by the size of the database, where both too few options and too many can potentially discourage the user to complete their registration. With few options, we wanted to counter this by having a comment section for food items they could not find.

Both data collection methods are self-administered which opens for the possibility of social desirability bias as participants might underreport unhealthy foods and overreport healthy foods. Recall bias might also be present in both the screener used for the diet quality scores and the 24-hour recall, causing differential measurement error although it is difficult to think of a mechanism in which this would explain the lack of evidence for an effect seen in our study.

Our estimates for the meeting micronutrient requirement outcomes are susceptible to misclassification bias. However, triangulating the closeness of the effect estimates to the null value, the consistency across micronutrients and the lack of evidence for an effect on the continuous micronutrient outcomes which cannot be caused by misclassification, it seems unlikely that misclassification bias would alter our results sufficiently to change the general findings from our study.

Using a DQS has its own strengths and limitations. It is a good way of assessing diet quality as it provides a straightforward path towards evaluating different aspects of an individuals’ diet [27], as well as creating a scale that is comparable to other studies. The DQS is based only on frequency and does not estimate portion sizes, leading to a lack of information regarding macro- and micronutrient intake. However, they provide a consistent method for assessing diet over time and are sensitive to dietary changes in intervention trials [58].

## Conclusion

As the first digital intervention trial of this kind, the PREPARED study did not find any significant improvement in diet at the end of the six months intervention. There was no evidence of having a healthier diet in any of the groups, and there were no differences in proportions reaching average requirement for selected micronutrients. The significant loss to follow up was a major limitation to identifying any effect of the intervention. Together with the limited engagement with the digital resource, a larger sample might be needed to identify the smaller possible changes in diet invoked by digital interventions in highly educated individuals such as that studied here. Future work focuses on reaching populations that might benefit more from this type of intervention, and new strategies for recruitment and keeping retention rates high needs to be developed.

## Supplementary Information

Supplementary Material 1.

## Data Availability

The datasets used and/or analysed during the current study are available from the corresponding author on reasonable request.
