# Supplementary material for: Effect of a digital dietary intervention on diet quality in young adults in Norway – The PREPARED randomised controlled trial
Source: BMC Nutr. 2026 Feb 21;12:83. doi: 10.1186/s40795-026-01278-4 (PMC13134278; doi:10.1186/s40795-026-01278-4)
Supplement: Supplementary file 1 — Supplementary Material 1. [file 40795_2026_1278_MOESM1_ESM.docx]

# Supplementary files

**Supplementary table S1.** See next page.

**Supplementary table S2.** Percentage reaching average requirement (AR)

| **Micronutrient adequacy** | | | | |
| --- | --- | --- | --- | --- |
|  | | % reaching average requirement | |  |
|  | NNR AR^1^ | Intervention (*n*=69) | Control (*n*=82) | *p*-value |
| Calcium | 750 mg/day | 46.4 | 43.9 | 0.77 |
| Folate | 250 μg/day | 52.2 | 54.9 | 0.92 |
| Iodine | 120 μg/day | 31.9 | 31.7 | 0.91 |
| Iron | 9/7 mg/day^2^ | 50.7 | 54.3 | 0.74 |
| Vitamin B12 | 3.2 μg/day | 59.4 | 57.3 | 0.87 |
| Vitamin C | 75/90 mg/day^2^ | 58.0 | 63.0 | 0.80 |
| Vitamin D | 7.5 μg/day | 43.5 | 39.0 | 0.89 |

^1^Cut-off values for reaching average requirement defined in the Nordic Nutrition Recommendations.

^2^Separate recommendations for females/males

**Supplementary table S3.** Estimated effect of PREPARED intervention on total diet score at 6 months.

| **Total diet score** | **Mean Difference**  **(Intervention – control)** | **(95% CI)** | ***p-*value** |
| --- | --- | --- | --- |
| Unadjusted | +0.4 | (-3.6, 4.3) | 0.86 |
| Adjusted* | +1.0 | (-3.0, 4.9) | 0.63 |

*Adjusted for educational level.

| **Diet Quality Score components**  Screener variable(s) | **Scoring Valence** | **Criteria for min score (0)** | **Criteria for max score (10)** | **‘MyFoodMonth 1.1’ Diet Quality Score scorings** | | | | | | | | | | | |
| --- | --- | --- | --- | --- | --- | --- | --- | --- | --- | --- | --- | --- | --- | --- | --- |
|  |  |  |  | **Never** | **1 a month** | **2-3 a month** | **1 a week** | **2-4 a week** | **5-6 a week** | **1 a day** | **2-3 a day** | | **4-5 a day** | | **≥6 a day** |
| **Vegetables**  Vegetables, including salad, cabbage, carrot, green beans, etc. (not potatoes or sweet potatoes) | positive | ≤1 x month | ≥4 x day | 0  (*n=*9) | | 1  (*n=*13) | 2  (*n=*37) | 4  (*n=*209) | 6  (*n=*162) | 8  (*n=*395) | 9  (*n=*469) | | 10  (*n=*81) | | |
| **Fruits**  Fruit and berries, including fresh, frozen, and canned (not juice or smoothie) | positive | ≤1 x month | ≥2 x day | 0  (*n=*104) | | 1  (*n=*107) | 2  (*n=*128) | 4  (*n=*307) | 6  (*n=*120) | 8  (*n=*250) | 10  (*n=*359) | | | | |
| **Grains (products)**  Cereal and porridge, Unsweetened (e.g., 4-Korn muesli, oatmeal, Go’dag muesli, and Weetabix)  Whole grain bread, crispbread, rolls (>50% whole grain), Whole grain dinner products (e.g., barley, pasta, couscous) | positive | ≤1 x month | 2-5 x day | 0  (*n=*27) | | 1  (*n=*18) | 2  (*n=*64) | 4  (*n=*167) | 6  (*n=*103) | 8*  (*n=*591) | 10  (*n=*405) | | | | 8* |
| **Fish**  Fatty fish and fish products (e.g., salmon, mackerel)  Lean fish and fish products (e.g., cod, pollock)  Fish spread (e.g., mackerel in tomato sauce) | positive | 0 | ≥1 x week | 0  (*n=*161) | 4  (*n=*78) | 7  (*n=*188) | 9  (*n=*307) | 10  (*n=*641) | | | | | | | |
| **Legumes**  Beans, lentils, chickpeas, peas (not green beans) | positive | 0 | ≥2 x day | 0  (*n=*172) | 1  (*n=*149) | 2  (*n=*207) | 4  (*n=*235) | 6  (*n=*360) | 8  (*n=*97) | 9  (*n=*119) | 10  (*n=*36) | | | | |
| **Meat (processed and red)**  Red meat, minced or cuts (beef, lamb, pork, goat)  Processed meat (e.g., bacon, spread, sausage) | negative | ≥2 x day | ≤1 x month | 10  (*n=*298) | | 8  (*n=*85) | 6  (*n=*256) | 4  (*n=*309) | 2  (*n=*208) | 1  (*n=*173) | 0  (*n=*46) | | | | |
| **Unsalted nuts and seeds**  Unsalted nuts and seeds | positive | 0 | 1-3 x day | 0  (*n=*172) | 1  (*n=*149) | 2  (*n=*207) | 4  (*n=*235) | 6*  (*n=*362) | 8*  (*n=*100) | 10  (*n=*150) | | 8* | | 6* | |
| **Sugar-sweetened beverages**  Sugar-sweetened beverages, Sugar-sweetened energy drinks (e.g., Gatorade, Red Bull) | negative | ≥1 x day | 0 | 10  (*n=*471) | 9  (*n=*222) | 8  (*n=*180) | 6  (*n=*175) | 4  (*n=*160) | 1  (*n=*56) | 0  (*n=*111) | | | | | |
| **Salty snacks**  Salty snacks (e.g., popcorn, chips, salty nuts) | negative | ≥2 x day | 0 | 10  (*n=*56) | 9  (*n=*120) | 8  (*n=*274) | 6  (*n=*477) | 4  (*n=*372) | 2  (*n=*29) | 1  (*n=*35) | 0  (*n=*12) | | | | |
| **Sugary foods**  Cereal and porridge, Sweetened (e.g., Special K, Corn Flakes with honey); Candy, including chocolate; Waffles, buns, cake, biscuits etc.; Ice cream, panna cotta, pudding, mousse, etc. | negative | ≥1 x day | 0 | 10  (*n=*18) | 9  (*n=*36) | 8  (*n=*50) | 6  (*n=*416) | 4  (*n=*453) | 1  (*n=*142) | 0  (*n=*260) | | | | | |
| *Participants’ correct categorisation could not be identified based on their score. The score could be the same regardless of whether they had higher or lower intake than what gave the optimal score. | | | | | | | | | **Total Possible Points:** | | | | | | **100** |

**Supplementary table S1.** ’MyFoodMonth 1.1’ Diet Quality Score scorings at baseline. The healthy components vegetables through unsalted nuts and seeds gave high scores on high intake, while the unhealthy components red and processed meats through sugary foods gave high scores with low consumption. Highlighted cells in whole grain and nuts symbolise grouped categories where the sample is contained in both cells.

**Estimating usual intake and understanding the impact of measurement error**

Our target for inference was usual micronutrient intake which can be defined as the long-term average daily consumption of a nutrient (Kirkpatrick et al., 2022) or expected intake after removal of short term day-day variation. However our analyses was based on short-term 24-hour recall.

To acknowledge this and gain an insight into the day-day variability of our estimates we used the subsample with repeated measurements to estimate the within- and between person variation for each micronutrient using multilevel models (N=514). These estimates were then used to calibrate or adjust the baseline intake for each person by removing the day-day component from the total variation. Modelling was done on a log-scale and then back-transformed to obtain the so-called usual intake for each person.

We applied this procedure to the baseline energy adjusted micronutrient values (per 10MJ) and non-energy adjusted. The non-adjusted usual intakes were used in a second step to calculate the sample prevalence meeting Norwegian AR, ie, prevalence accounting for day-day variation in measurement.

Table S4 shows the summary statistics for the distribution of each energy adjusted micronutrient intake at baseline for the single 24-hour recall and usual intake. Figure SX shows the distributions. Generally day-to-day variation in intake was greater than differences between individuals. This is reflected in the low ICCs and visualised when comparing the distributions of usual v single 24 hour intake (figure S1). The medians for each usual micronutrient intake were smaller and the IQR much narrower, reflecting the substantial shrinkage of measures when day-day variation was removed (Table S4).

**Table S4**. Median (interquartile range) for energy adjusted 24 hour and usual* micronutrient intake at baseline (n=733). Also shown are the variance components and intraclass correlation (ICC)

|  |  | Measure: | | Variance components (log(Y)): | |  |
| --- | --- | --- | --- | --- | --- | --- |
|  |  | Single 24 hour | Usual (calibrated) | Between-person | Within-person | ICC |
| Calcium | mg per 10MJ | 1212 (815, 1847) | 1096 (971, 1223) | 0.08 | 0.26 | 0.23 |
| Folate | μg per 10MJ | 403 (277, 621) | 392 (341, 464) | 0.11 | 0.21 | 0.35 |
| Iodine | μg per 10MJ | 157 (96, 255) | 146 (127, 167) | 0.12 | 0.44 | 0.22 |
| Iron | mg per 10MJ | 15.2 (11.1, 21.5) | 14.9 (13.3, 17.1) | 0.10 | 0.21 | 0.33 |
| Vitamin B12 | μg per 10MJ | 7.5 (4.5, 12.0) | 6.7 (5.5, 8.0) | 0.29 | 0.67 | 0.30 |
| Vitamin C | mg per 10MJ | 152 (72, 278) | 122 (93, 150) | 0.59 | 1.39 | 0.30 |
| Vitamin D | μg per 10MJ | 6.5 (2.2, 15.7) | 5.6 (3.9, 8.0) | 1.30 | 3.41 | 0.28 |


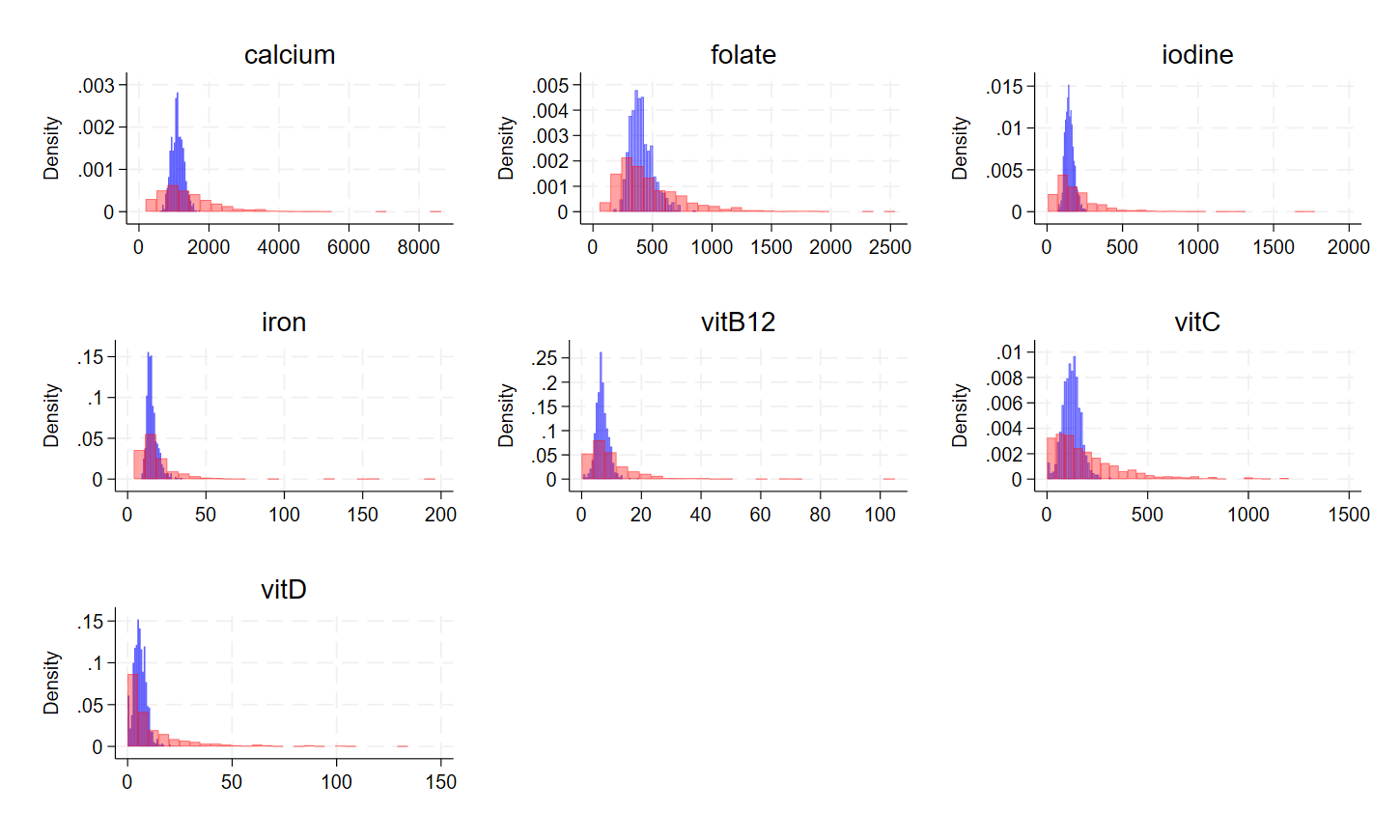


**Figure S1**. Distribution of measured micronutrient intakes at baseline from 24-hour diet recall (red) and estimated usual intake (blue) after removal of noise from day-to-day variation. Values are energy adjusted per 10 MJ.

The prevalences for meeting Norwegian micronutrient AR guidelines are shown in Table S5 for both the single 24-hour dietary recall and the calibrated usual intake after removing day-to-day variation. The estimated prevalences from usual intake differed in both directions. Vitamin D showed greatest disparity between prevalences estimated from single and usual intake. This might reflect the fact it less frequently consumed compared to others nutrient rendering the modelling strategy and frequency of data collection inappropriate for estimating usual intake for this vitamin.

**Table S5.** Median (interquartile range) for energy adjusted 24 hour and usual* micronutrient intake at baseline (n=733). Also shown are the variance components and intraclass correlation (ICC)

|  | Meeting Norwegian AR (%): | |
| --- | --- | --- |
|  | Single 24 hour | Usual (calibrated) |
| Calcium | 53.1 | 41.0 |
| Folate | 54.0 | 53.7 |
| Iodine | 43.0 | 23.1 |
| Iron | 60.3 | 65.5 |
| Vitamin B12 | 70.2 | 80.5 |
| Vitamin C | 59.2 | 54.4 |
| Vitamin D | 33.4 | 7.0 |

Figure S2 shows the results from the secondary analysis of continuous micronutrient intake. There was no evidence of a distribution-wide shift in any of the micronutrients caused by the Prepared intervention.


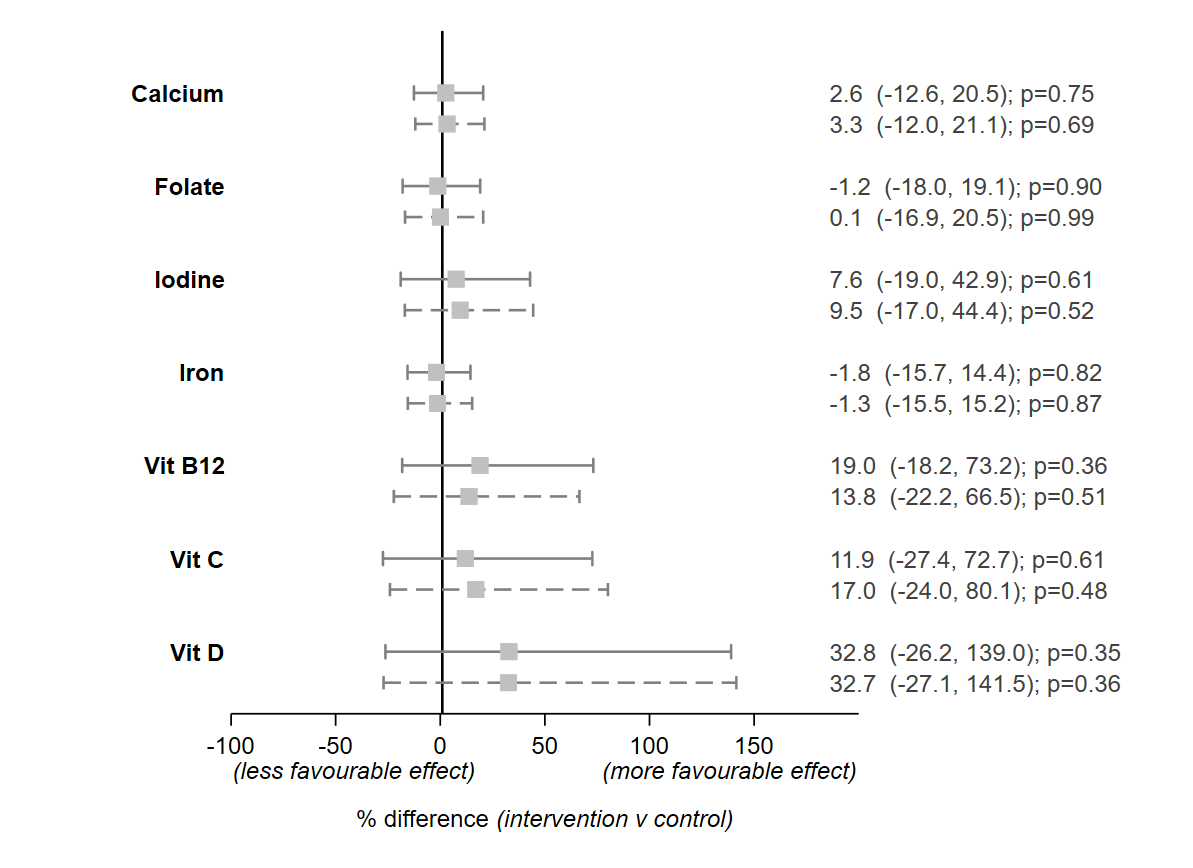


**Figure S2** Estimated effects of intervention on logged micronutrient levels. Effects have been back transformed and converted into % differences between groups. The solid lines are the unadjusted effects and the dashed adjusted for education. The estimates and 95% CI are listed on the right-hand side of the plot (n=150).
